# Recommended reference values for serum lipids during early and middle pregnancy: a retrospective study from China

**DOI:** 10.1186/s12944-018-0885-3

**Published:** 2018-10-31

**Authors:** Chen Wang, Lingying Kong, Yide Yang, Yumei Wei, Weiwei Zhu, Rina Su, Li Lin, Huixia Yang

**Affiliations:** 1 Department of Obstetrics and Gynecology of Peking University First Hospital, Beijing, China; 2 Teaching and Researching office of Child and Adolescent Health, School of Medicine, Hunan Normal University, Changsha, China; 3 National Institute of Hospital Administration, Beijing, China; 4 Peking University First Hospital, Xianmen Street No. 1, Xicheng District, Beijing, 100034 China

**Keywords:** Total cholesterol (TC), Triglycerides (TG), High-density lipid cholesterol (HDL-C), Low-density lipid cholesterol (LDL-C), Reference range, Pregnancy

## Abstract

**Background:**

Disturbances in maternal lipid metabolism have been shown to increase the risk of adverse pregnancy outcomes. However, there is no consensus as to what constitutes normal maternal lipid values during pregnancy. Thus, the aim of this study was to establish serum lipid reference ranges during early and middle pregnancy.

**Methods:**

We conducted a retrospective survey in Beijing from 2013 to 2014. A total of 17,610 singleton pregnancies with lipid data from early and middle pregnancy were included. First, after excluding women with adverse pregnancy outcomes, we performed a descriptive analysis of total cholesterol (TC), triglycerides (TG), high-density lipid cholesterol (HDL-C) and low-density lipid cholesterol (LDL-C) levels using means and standard deviations to determine appropriate percentiles. Second, in the total population, we examined the lipid levels in different trimesters with the risk of adverse pregnancy outcomes using categorical analyses and logistic regression models. Third, we determined the lipid reference range in early and middle pregnancy based on the first two results. Finally, based on the reference ranges we determined, we assessed whether the number of abnormal lipid values affected the risk of adverse pregnancy outcomes.

**Results:**

(1) Serum levels of TC, TG, LDL-C and HDL-C all increased significantly from early to middle pregnancy, with the greatest increase in TG. (2) A trend towards an increasing incidence of adverse pregnancy outcomes was observed with increasing levels of TC, TG, and LDL-C and decreasing levels of HDL-C in both early and middle pregnancy. (3) We recommend that serum TC, TG and LDL-C reference values in early and middle pregnancy should be less than the 95th percentiles, whereas that of HDL-C should be greater than the 5th percentile, i.e., in early pregnancy, TC < 5.64 mmol/L, TG < 1.95 mmol/L, HDL-C > 1.23 mmol/L, and LDL-C < 3.27 mmol/L, and in middle pregnancy, TC < 7.50 mmol/L, TG < 3.56 mmol/L, HDL-C > 1.41 mmol/L, and LDL-C < 4.83 mmol/L. (4) Higher numbers out-of-range lipids during early and middle pregnancy were correlated with a higher risk of adverse pregnancy outcomes.

**Conclusions:**

The reference ranges recommended in this paper can identify pregnant women with unfavourable lipid values.

## Background

Pregnancy is a unique physiological state in which the mother’s metabolic functioning undergoes alterations throughout pregnancy to ensure adequate energy stores, including glucose, amino acids and lipids, for appropriate foetal growth and development. These adaptations include complex changes in maternal lipid metabolism. The primary characteristic features of lipid metabolism changes during pregnancy are fat accumulation, increased tissue lipolysis and maternal hyperlipidaemia [1, 2]. These changes are physiologically necessary [3], and the corresponding clinical manifestations are a continuous increase in levels of maternal lipid concentrations from preconception to the third trimester [2, 4, 5]. However, disturbances in maternal lipid metabolism have been shown to increase the risk of adverse pregnancy outcomes, including gestational diabetes mellitus (GDM), pre-eclampsia (PE), preterm birth and foetal growth disorders [6–10].

In the non-pregnant state, high serum concentrations of total cholesterol (TC), triglycerides (TG) and low-density lipid cholesterol (LDL-C), and a reduction in serum high-density lipoprotein (HDL-C), are amongst the features of dyslipidaemia related to metabolic syndrome, and they are associated with an increased risk of cardiovascular disease later in life [11, 12]. However, there is no consensus as to what constitutes normal maternal lipid values during pregnancy. Therefore, obstetricians often cannot determine whether lipid levels are normal for a given period of pregnancy. Thus, pregnant women with out-of-range lipid values cannot be recognized and provided appropriate risk reduction interventions in a timely fashion. Therefore, the aim of this paper was to fill this gap by describing blood lipid concentrations in early and middle pregnancy and examining their correlations with adverse pregnancy outcomes. We also discuss the recommended reference ranges for maternal blood lipid concentrations in early and middle pregnancy.

## Methods

### Data sources

This present analysis was part of a large retrospective study. In that study, 15 hospitals in Beijing including Peking University First Hospital (PUFH) were chosen as clusters by a systemic cluster sampling method based on their number of deliveries. A total of 15,194 pregnant women who delivered in these hospitals from 20 June to 30 November of 2013 were recruited. In addition, 4,072pregnant women who delivered from 1 December of 2013 to 30 November of 2014 at PUFH were also studied. Therefore, the total sample size was 19,266. This study was reviewed and approved by the Institutional Review Board of the First Hospital, Peking University (Reference number: 2013[572]). All participants provided written informed consent, and the ethics committee approved this consent procedure.

All participants in the study were eligible for the present analysis if they had a live-born singleton infant and data regarding pregnancy lipid profiles in early and middle pregnancy and pregnancy course and outcome. Some women in our study were excluded for one or more of the following reasons: pre-existing diabetes, hypertension, thyroid disease or immune system disorders, multiple births and missing data on major items such as pre-pregnancy weight, height, 75 g oral glucose tolerance test results, PE diagnosis, birth weight and gestational age. Overall, a total of 19,044 participants were available for and included in the final analysis.

### Data collection

A questionnaire was designed to gather demographic and medical information by interviewing all pregnant women who delivered during the study period and by extracting data from medical records the day following birth. Demographic information was collected and recorded during a face-to-face interview in the patient’s room; this information included maternal age (years), height (centimetres), education and pre-pregnancy weight. In addition, medical data, including lipid concentrations during pregnancy, gestational age, birth weight, and pregnancy complications (mainly the occurrence of pregnancy-induced-hypertension, PE and GDM), were extracted from each patient’s medical record.

All investigators in each hospital were trained before the survey was administered. Each completed questionnaire was verified by an inspector. Data were coded and entered into a specially designed data software program that automatically checked for out-of-range values and logical mistakes. All compiled data were entered by two persons independently and then verified by a third person.

### Definitions

1. GDM: According to the new criteria amended in August 2014 in China, a diagnosis of GDM should be made when any one value met or exceeded a 0-h glucose level of 5.1 mM, a 1-h glucose level of 10.0 mM, and a 2-h glucose level of 8.5 mM after a diagnostic 75-g OGTT between the 24th and 28th weeks of gestation. A 0-h glucose level of 7.0 mM or a 2-h glucose level of 11.1 mM was considered sufficient to diagnose DM at any time, regardless of pregnancy stage [13].
2. Pregnancy-induced hypertension: Pregnancy-induced hypertension included both gestational hypertension and PE. Gestational hypertension: defined as blood pressure elevation [systolic blood pressure ≥ 140 mmHg or diastolic blood pressure ≥ 90 mmHg] at > 20 weeks’ gestation in the absence of proteinuria [14]. PE: defined as new-onset hypertension (systolic blood pressure ≥ 140 mmHg or diastolic blood pressure ≥ 90 mmHg) and new-onset proteinuria (300 mg of protein in 24 h or a urine protein/creatinine ratio of 0.3 mg/dl) after 20 weeks of gestation, in a previously normotensive woman [14].
3. Pre-pregnancy body mass index (p-BMI) was calculated as pre-pregnancy weight (within 3 months before pregnancy) in kilograms divided by the square of the height in metres (kg/m^2^).
4. Preterm birth: Gestational age of less than 37 weeks at delivery.
5. Macrosomia: Foetal birth weight ≥ 4000 g, regardless of gestational age.
6. Large for-gestational-age (LGA): Newborn birthweight above the 90th percentile for gestational age in accordance with the international standards for sex-specific newborn size for each gestational age based on data from the Newborn Cross-Sectional Study subpopulation [15].

### Statistical analysis

The statistical analysis was performed using the SPSS 17.0 statistical software package (Peking University Clinical Research Institute). We first excluded those participants with overweight/obesity, GDM, pregnancy-induced hypertension, preterm birth, macrosomia and LGA from the analysis to see the normal change trend of maternal lipid levels during the first two trimesters of pregnancy by calculating the average blood lipid levels every 4 weeks. Additionally, descriptive analyses of TG, TC, HDL-C and LDL-C levels were generated using the means and standard deviations and appropriate percentiles for this healthy group in both early and middle pregnancy. Then, to establish the reference ranges of lipids in early and middle pregnancy, associations of maternal blood lipid levels in different trimesters with the risk of GDM, pregnancy-induced hypertension, PE, preterm birth, macrosomia, LGA and all factors combined were examined by categorical analyses and logistic regression models for the total sample. For the categorical analyses, each lipid measure was divided into five equal categories, with category 1 representing the bottom 20 percentiles and category 5 representing the top 20 percentiles. For the multivariate logistic regression analysis, odds ratios (ORs) and 95% confidence intervals (CIs) were calculated, and maternal age, p-BMI, educational level and gestational age at the time of lipid measurement were adjusted as confounders. Finally, based on the reference range we determined, we assessed whether the number of abnormal lipid values affected the risks of adverse pregnancy outcomes.

## Results

In total, 17,610 mother–newborn pairs were included in our study. There were 3678 mothers (20.9%) diagnosed with GDM, 705 (4.0%) diagnosed with pregnancy-induced hypertension, and 361 (2.0%) diagnosed with PE. The newborns in this study had a mean gestational age of 39.4 ± 1.5 weeks, and their mean birth weight was 3376 ± 468 g. There were 804 (4.6%) babies born prematurely, 3116 newborns (17.7%) with LGA, and 1499 newborns (8.5%) with macrosomia. After excluding those mother–newborn pairs with one or more of the adverse outcomes, 11,566 healthy mothers and their newborns remained. Their age and p-BMI levels were all significantly lower than those with adverse outcomes (Table 1).

**Table 1:** Maternal baseline characteristics

	Total	Healthy	With adverse outcomes	p
	*N* = 19,044	*N* = 11,566	*N* = 7478	
Age (year)	29.5 ± 4.1	29.1 ± 3.9	30.1 ± 4.2	< 0.001
p-BMI (kg/m2)	21.3 ± 4.7	20.7 ± 4.5	22.2 ± 5.0	< 0.001
< 18.5 (%)	2886 (16.5)	2132 (20.0)	754 (11.0)	
18.5–23.9 (%)	11,058 (63.1)	6988 (65.4)	4070 (59.6)	
24–27.9 (%)	2639 (15.1)	1203 (11.3)	1436 (21.0)	
≥ 28 (%)	928 (5.3)	359 (3.4)	569 (8.3)	
Education level				0.396
College or higher (%)	12,766 (73.2)	7786 (73.1)	4980 (73.3)	
Up to high school (%)	4683 (26.8)	2866 (26.9)	1817 (26.7)	

Continuous variables were expressed as the means ± SD and categorical variables were expressed as n (%)

Abbreviations: *p-BMI* pre-pregnancy body mass index (calculated as weight in kilograms divided by the square of height in metres)

*P* value indicates a significant difference between the healthy group and the adverse outcomes group

**Fig. 1:**
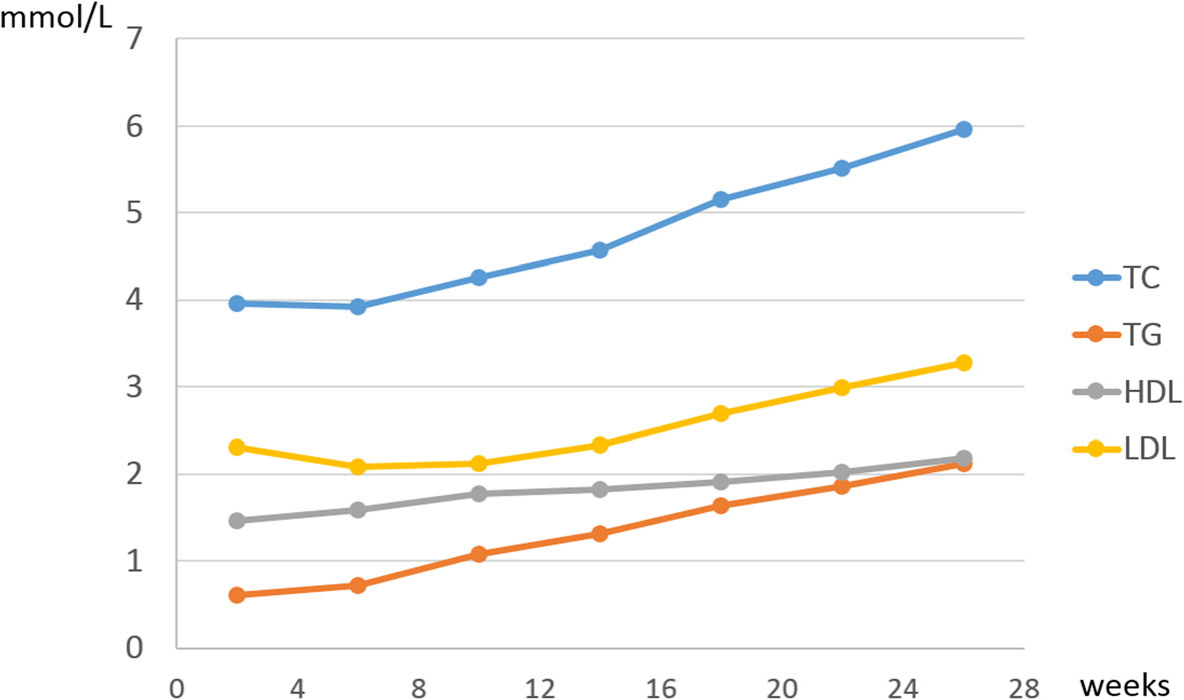
The changing curve of maternal blood lipids during pregnancy (Abbreviations: TC, total cholesterol; TG, triglycerides; HDL-C, high-density lipid cholesterol; LDL-C, low-density lipid cholesterol)

Figure 1 shows the normal trend change of maternal lipid levels in early and middle pregnancy by presenting the average blood lipid levels for every 4 weeks in women with no adverse pregnancy outcomes. In addition, targeting this subgroup, Table 2 shows the mean concentrations and the 2.5th, 5th, 10th, 25th, 50th, 75th, 90th, 95th and 97.5th percentiles for TC, TG, HDL-C and LDL-C in early and middle pregnancy. Serum concentrations of TC, TG, HDL-C and LDL-C were significantly higher in pregnant women in the second trimester than they were in the first trimester. The most prominent change was a 2.1-fold TG elevation in late pregnancy. Specifically, from the second to first trimester, TC, TG, HDL-C and LDL-C concentrations had 1.38, 2.10, 1.12 and 1.58-fold elevations, respectively.

**Table 2:** Serum lipids in pregnant women

	Trimester	x¯ ± s	Percentile								
			2.5%	5%	10%	25%	50%	75%	90%	95%	97.5%
TC	First (*n* = 6802)	4.35 ± 0.77	3.01	3.2	3.44	3.83	4.29	4.8	5.32	5.64	5.97
	Second(*n* = 2878)	5.62 ± 1.09	3.76	4.01	4.29	4.84	5.53	6.36	7.07	7.50	7.87
TG	First (*n* = 6827)	1.09 ± 0.6	0.4	0.46	0.54	0.74	1.00	1.31	1.67	1.95	2.3
	Second (*n* = 2881)	1.96 ± 0.83	0.85	0.95	1.09	1.37	1.81	2.36	3.00	3.56	4.04
HDL	First (*n* = 6820)	1.8 ± 0.41	1.14	1.23	1.34	1.53	1.76	2.02	2.28	2.45	2.59
	Second (*n* = 2881)	2.03 ± 0.43	1.32	1.41	1.54	1.75	2.00	2.29	2.55	2.72	2.88
LDL	First (*n* = 6818)	2.25 ± 0.6	1.27	1.4	1.56	1.86	2.2	2.58	3.00	3.27	3.54
	Second (*n* = 2819)	3.14 ± 0.92	1.69	1.86	2.07	2.47	3.00	3.71	4.41	4.83	5.22

Abbreviations: *TC* total cholesterol, *TG* triglycerides, *HDL-C* high-density lipid cholesterol, *LDL-C* low-density lipid cholesterol

**Fig. 2:**
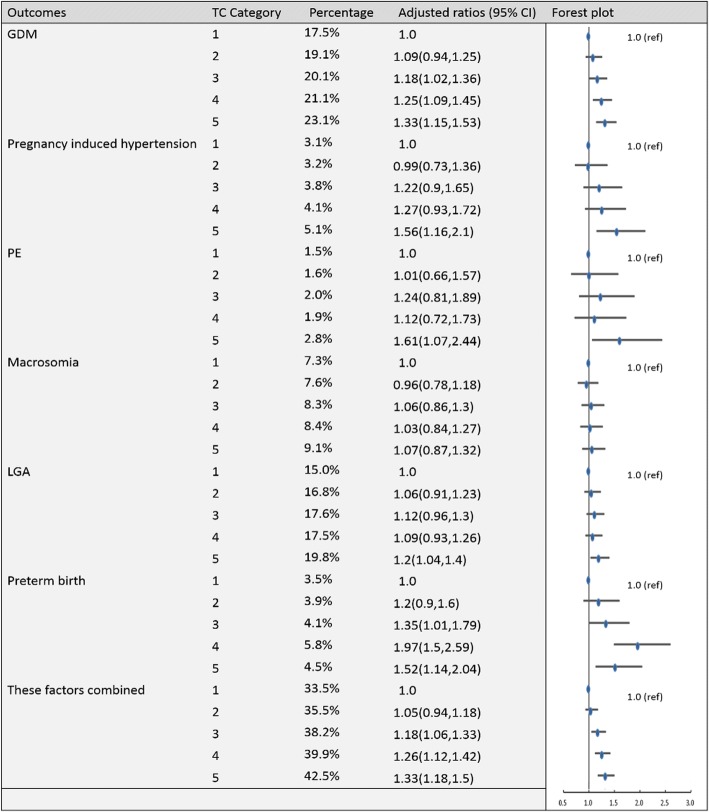
Categorical analyses of the risk of TC in early pregnancy and adverse pregnancy outcomes (Abbreviations: HDL-C, high-density lipid cholesterol; GDM, gestational diabetes mellitus; PE, preeclampsia; LGA, large for gestational age; Adjusted for maternal age, pre-pregnancy body mass index, educational level and gestational age at the time of lipid measurement)

**Fig. 3:**
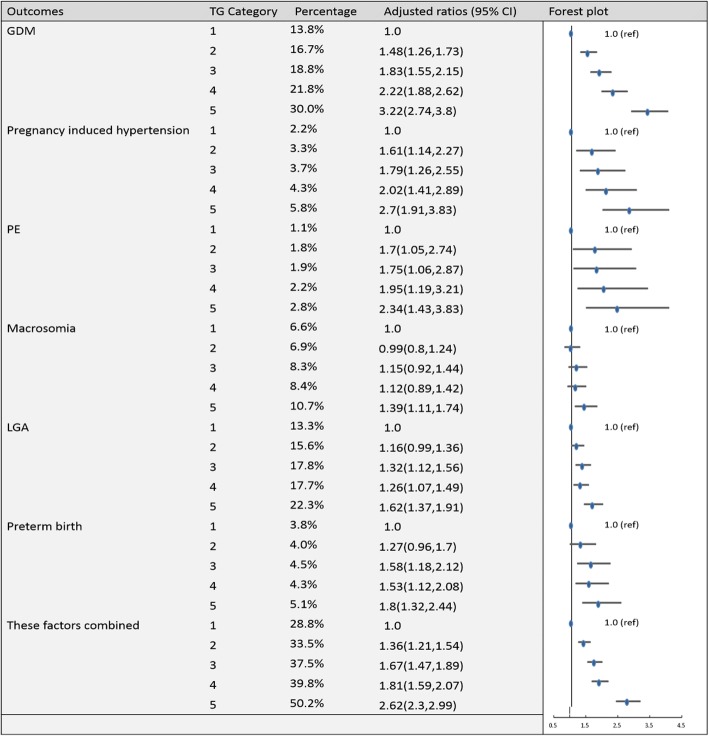
Categorical analyses of the risk of TG in early pregnancy and adverse pregnancy outcomes (Abbreviations: HDL-C, high-density lipid cholesterol; GDM, gestational diabetes mellitus; PE, preeclampsia; LGA, large for gestational age; Adjusted for maternal age, pre-pregnancy body mass index, educational level and gestational age at the time of lipid measurement)

**Fig. 4:**
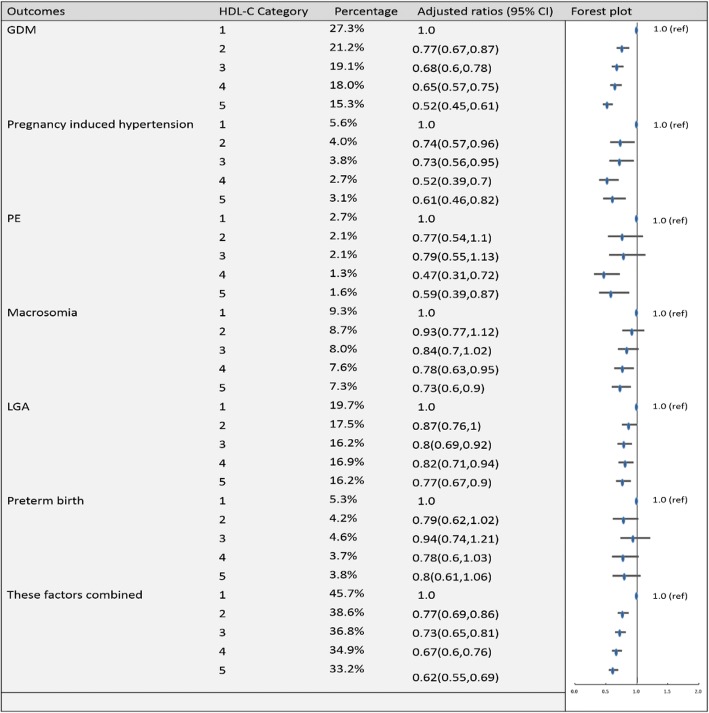
Categorical analyses of the risk of HDL-C in early pregnancy and adverse pregnancy outcomes (Abbreviations: HDL-C, high-density lipid cholesterol; GDM, gestational diabetes mellitus; PE, preeclampsia; LGA, large for gestational age; Adjusted for maternal age, pre-pregnancy body mass index, educational level and gestational age at the time of lipid measurement)

**Fig. 5:**
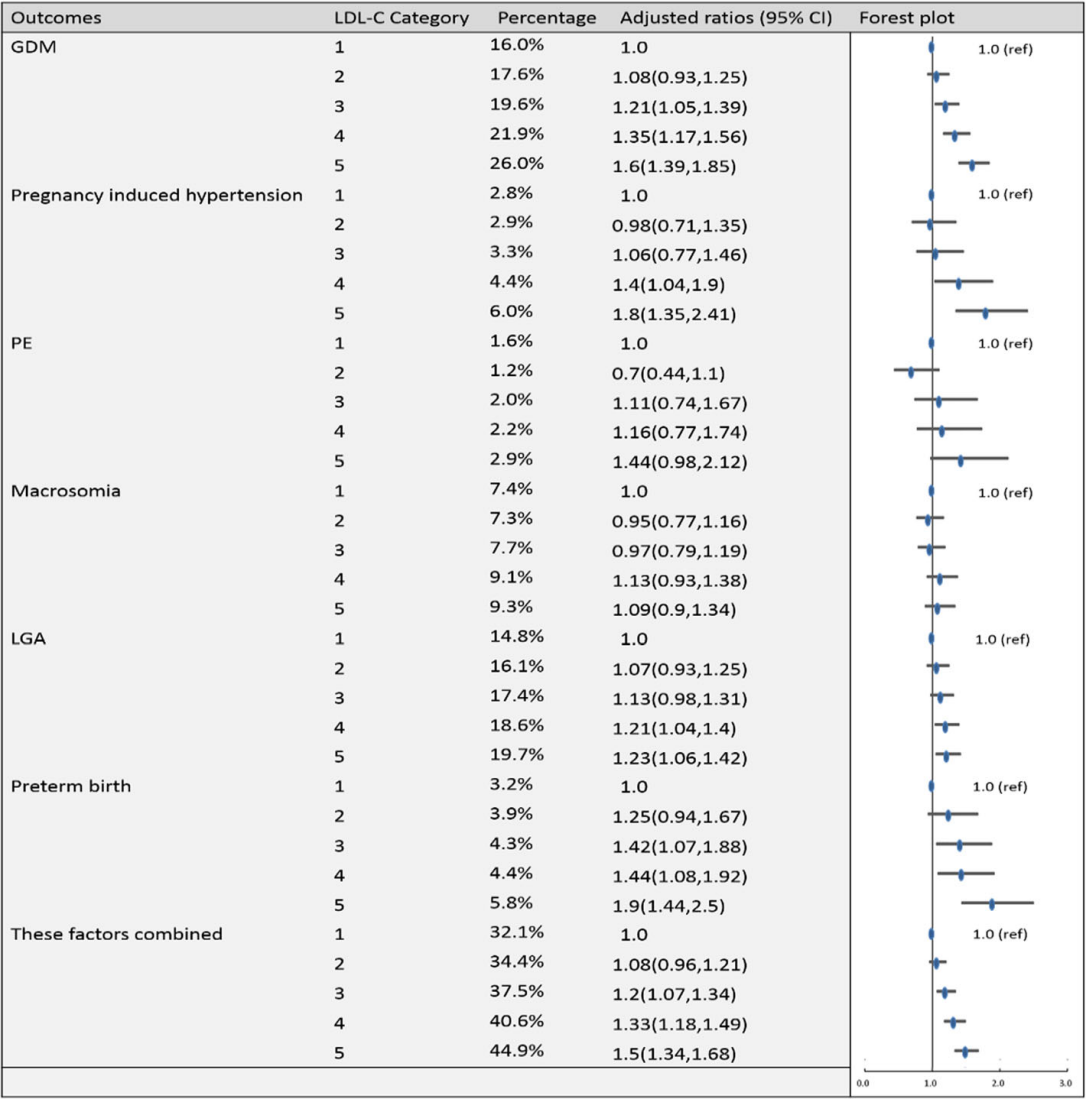
Categorical analyses of the risk of LDL-C in early pregnancy and adverse pregnancy outcomes (Abbreviations: HDL-C, high-density lipid cholesterol; GDM, gestational diabetes mellitus; PE, preeclampsia; LGA, large for gestational age; Adjusted for maternal age, pre-pregnancy body mass index, educational level and gestational age at the time of lipid measurement)

**Fig. 6:**
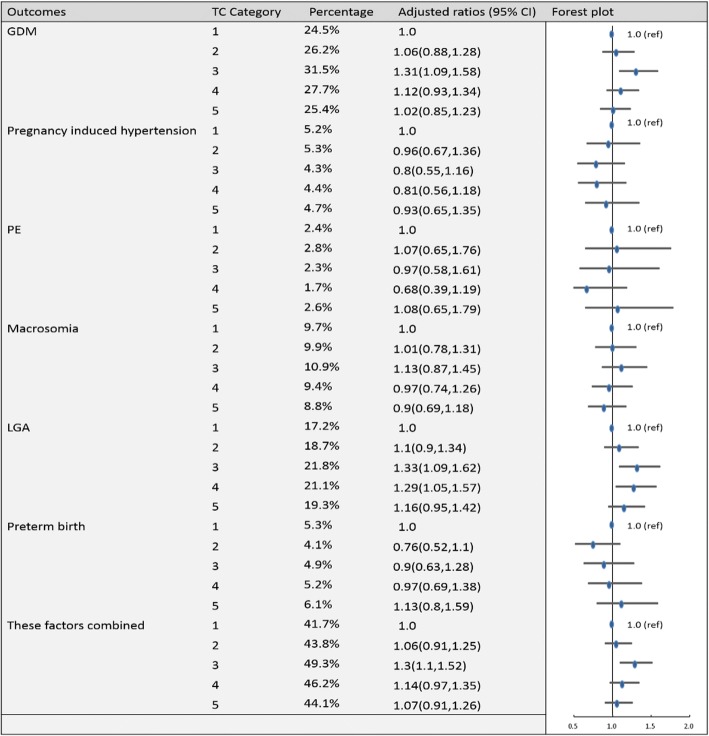
Categorical analyses of the risk of TC in middle pregnancy and adverse pregnancy outcomes (Abbreviations: HDL-C, high-density lipid cholesterol; GDM, gestational diabetes mellitus; PE, preeclampsia; LGA, large for gestational age; Adjusted for maternal age, pre-pregnancy body mass index, educational level and gestational age at the time of lipid measurement)

**Fig. 7:**
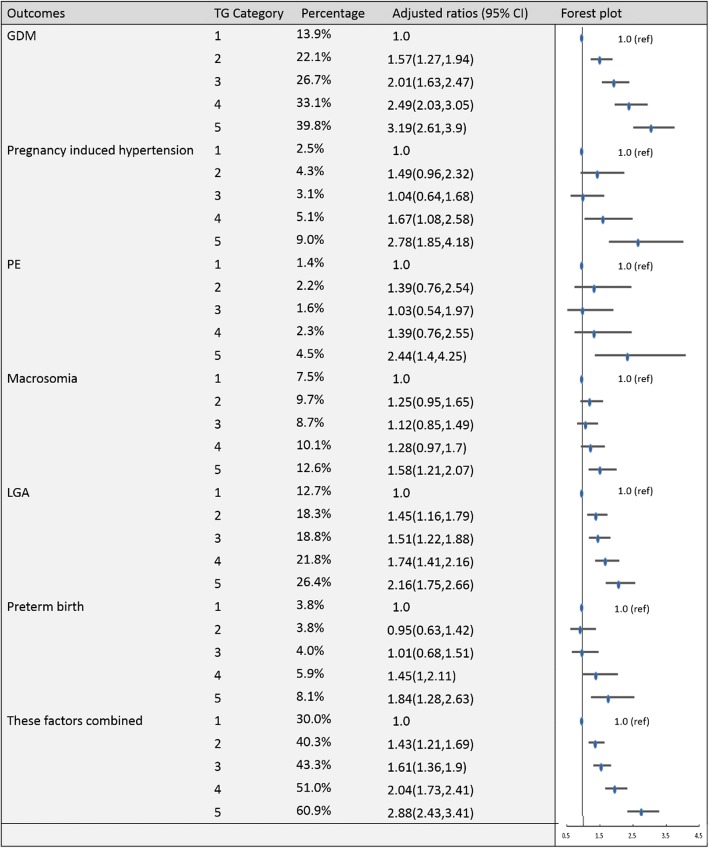
Categorical analyses of the risk of TG in middle pregnancy and adverse pregnancy outcomes (Abbreviations: HDL-C, high-density lipid cholesterol; GDM, gestational diabetes mellitus; PE, preeclampsia; LGA, large for gestational age; Adjusted for maternal age, pre-pregnancy body mass index, educational level and gestational age at the time of lipid measurement)

**Fig. 8:**
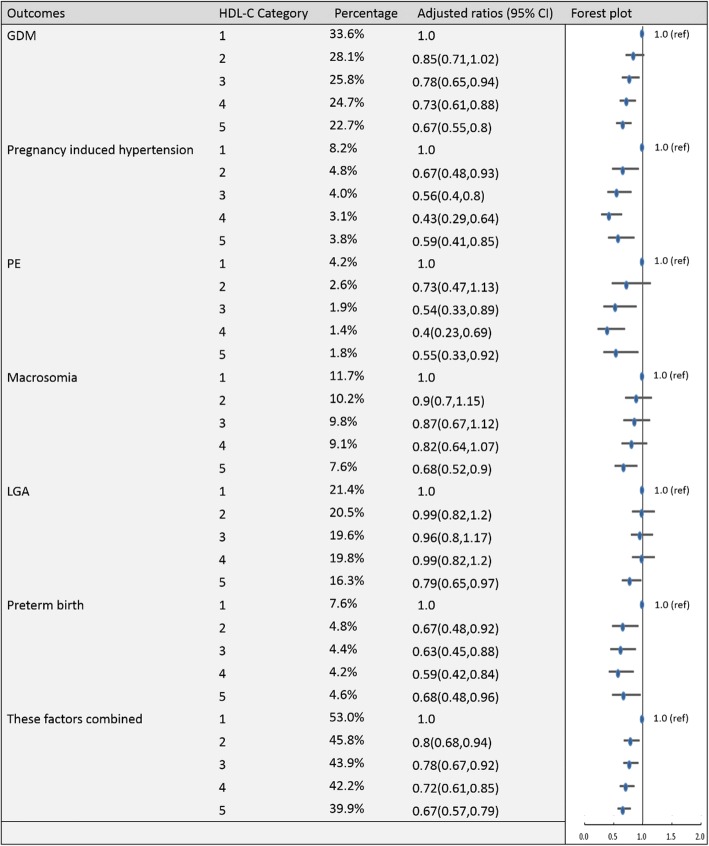
Categorical analyses of the risk of HDL-C in middle pregnancy and adverse pregnancy outcomes (Abbreviations: HDL-C, high-density lipid cholesterol; GDM, gestational diabetes mellitus; PE, preeclampsia; LGA, large for gestational age; Adjusted for maternal age, pre-pregnancy body mass index, educational level and gestational age at the time of lipid measurement)

**Fig. 9:**
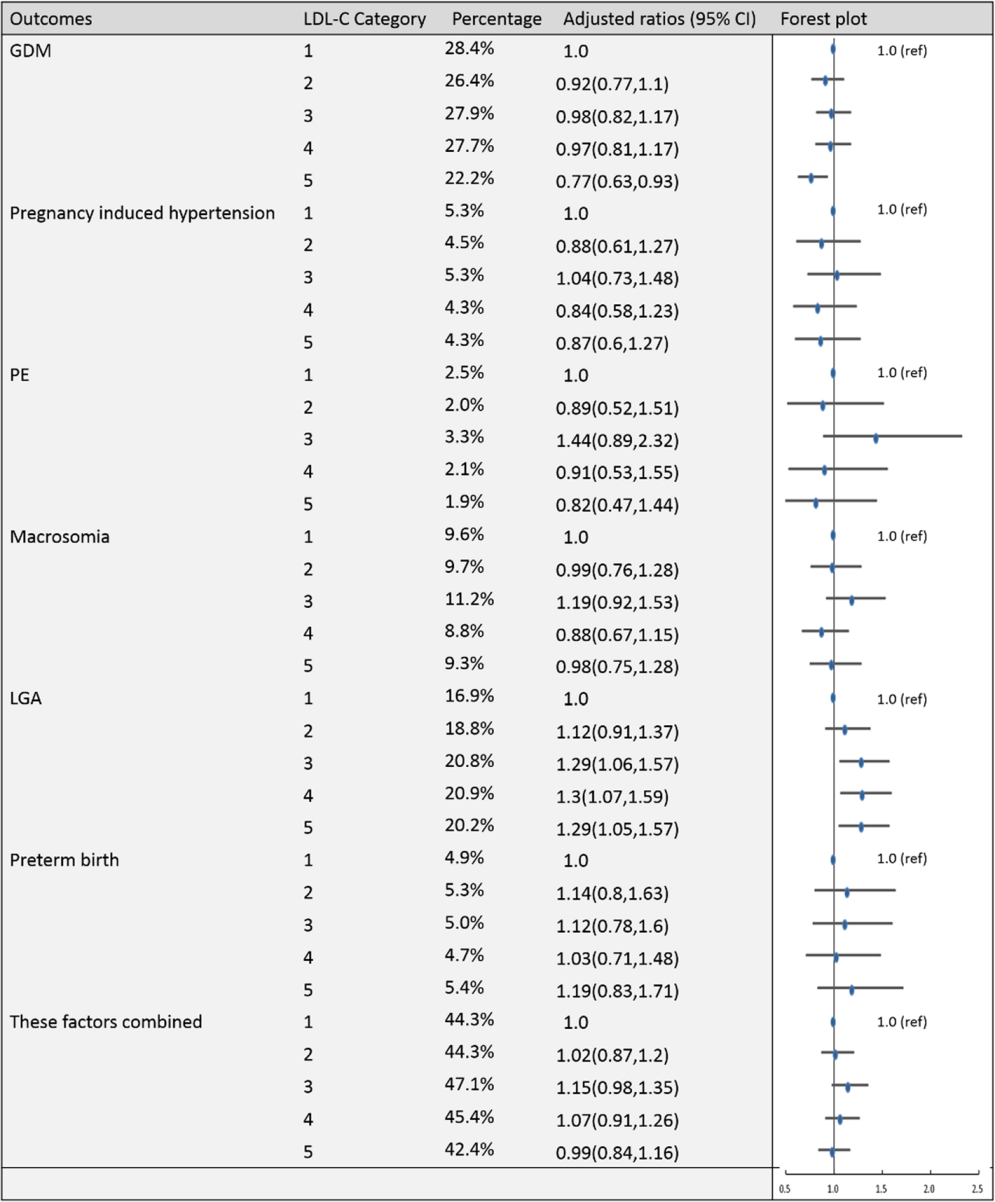
Categorical analyses of the risk of LDL-C in middle pregnancy and adverse pregnancy outcomes (Abbreviations: HDL-C, high-density lipid cholesterol; GDM, gestational diabetes mellitus; PE, preeclampsia; LGA, large for gestational age; Adjusted for maternal age, pre-pregnancy body mass index, educational level and gestational age at the time of lipid measurement)

To establish the reference ranges of lipids in early and middle pregnancy, we then examined the associations of maternal blood lipid levels in different trimesters with the risk of GDM, pregnancy-induced hypertension, PE, preterm birth, macrosomia, LGA and these factors combined for the entire sample. Figures 2, 3, 4, 5, 6, 7, 8 and 9 show that the frequencies of GDM, pregnancy-induced hypertension, PE, preterm birth, macrosomia and LGA increased as TC, TG and LDL-C levels increased and decreased as HDL-C levels increased in early and middle pregnancy. In the multivariable adjusted model, we observed that for the associations between maternal early pregnancy lipid profiles and adverse pregnancy outcomes, every unit increase in TC, TG and LDL increased the risk of GDM, pregnancy-induced hypertension, PE, preterm birth and LGA, and every mmol/L elevation in TG concentration was associated with an increased risk of macrosomia. By contrast, every unit increase in HDL-C reduced the risk of GDM, pregnancy-induced hypertension, macrosomia and LGA. Furthermore, for the associations between maternal middle pregnancy lipid profiles and adverse pregnancy outcomes, we discovered that every unit increase in TG increased the risk of GDM, pregnancy-induced hypertension, PE, preterm birth, macrosomia and LGA, and every mmol/L elevation in TC and LDL-C increased the risk of preterm birth and LGA, respectively. In addition, every unit increase in HDL-C reduced the risk of GDM, pregnancy-induced hypertension, PE and preterm birth (Table 3). Therefore, we believe that higher levels of TC, TG, LDL-C and lower levels of HDL-C are worse in both the first trimester and the second trimester. Thus, because the normal reference range is defined as 95th percentiles of the distributions, we recommend that the reference values of serum TC, TG and LDL in early and middle pregnancy should be less than the 95th percentile and that of HDL should be greater than the 5th percentile. Specifically, the recommended reference values for serum lipids in early pregnancy should be: TC < 5.64 mmol/L, TG < 1.95 mmol/L, HDL-C > 1.23 mmol/L and LDL-C < 3.27 mmol/L, and the recommended reference values for serum lipids in middle pregnancy should be: TC < 7.50 mmol/L, TG < 3.56 mmol/L, HDL-C > 1.41 mmol/L and LDL-C < 4.83 mmol/L.

**Table 3:** Logistic regression analysis of the risk of lipids and adverse pregnancy outcomes

	GDM	Pregnancy-induced hypertension	PE	Macrosomia	LGA	Preterm birth	Total
Early pregnancy							
TC	1.137 (1.073–1.204)	1.199 (1.069–1.344)	1.190 (1.018–1.391)	1.028 (0.946–1.117)	1.081 (1.108–1.148)	1.183 (1.106–1.318)	1.149 (1.095–1.205)
TG	1.465 (1.372–1.564)	1.270 (1.150–1.403)	1.236 (1.082–1.412)	1.156 (1.064–1.257)	1.177 (1.103–1.255)	1.147 (1.031–1.276)	1.455 (1.365–1.551)
HDL	0.621 (0.550–0.700)	0.710 (0.556–0.907)	0.773 (0.555–1.076)	0.724 (0.612–0.858)	0.806 (0.715–0.907)	0.963 (0.781–1.186)	0.720 (0.655–0.792)
LDL	1.254 (1.174–1.339)	1.370 (1.220–1.537)	1.123 (1.005–1.256)	1.046 (0.963–1.135)	1.073 (1.008–1.143)	1.167 (1.052–1.293)	1.234 (1.166–1.307)
Middle pregnancy							
TC	1.003 (0.951–1.059)	1.021 (0.915–1.139)	1.012 (0.868–1.179)	0.953 (0.882–1.030)	1.038 (0.980–1.099)	1.151 (1.040–1.274)	1.1033 (0.985–1.083)
TG	1.428 (1.344–1.516)	1.313 (1.195–1.442)	1.211 (1.063–1.379)	1.123 (1.039–1.214)	1.220 (1.149–1.296)	1.225 (1.115–1.345)	1.430 (1.349–1.516)
HDL	0.746 (0.650–0.857)	0.577 (0.430–0.773)	0.601 (0.400–0.903)	0.842 (0.692–1.024)	0.906 (0.785–1.046)	0.752 (0.576–0.983)	0.780 (0.793–0.879)
LDL	0.902 (0.844–1.000)	0.995 (0.872–1.135)	0.982 (0.817–1.181)	0.954 (0.869–1.047)	1.079 (1.008–1.155)	1.090 (0.965–1.230)	0.987 (0.982–1.044)

Abbreviations: *TC* total cholesterol, *TG* triglycerides, *HDL-C* high-density lipid cholesterol, *LDL-C* low-density lipid cholesterol, *GDM* gestational diabetes mellitus, *PE* preeclampsia, *LGA* large for gestational age

Adjusted for maternal age, pre-pregnancy body mass index, educational level and gestational age at the time of lipid measurement

Finally, based on the reference values we determined, we further divided the entire sample into five groups: Group A: no out-of-range lipid profiles; Group B: one out-of-range lipid; Group C: two out-of-range lipids; and Group D: three or four out-of-range lipids. As shown in Tables 4 and 5, the prevalence of GDM, pregnancy-induced hypertension, PE, preterm birth, macrosomia and LGA increased with increased numbers of out-of-range lipids in early pregnancy, and pregnancy-induced hypertension, PE and preterm birth increased with increased numbers of out-of-range lipids in middle pregnancy. In the first trimester, after adjusting for confounders, Group B had a significantly higher incidence of GDM, pregnancy-induced hypertension, PE, macrosomia and LGA than Group A; Group C had a significantly higher incidence of GDM, pregnancy-induced hypertension, PE, LGA and preterm birth than Group A; and Group D had a significantly higher incidence of GDM, pregnancy-induced hypertension and LGA than Group A. In middle pregnancy, Group B had a significantly higher incidence of GDM and pregnancy-induced hypertension than Group A; Group C had a significantly higher incidence of GDM, pregnancy-induced hypertension and preterm birth than Group A; and Group D had a significantly higher incidence of preterm birth than Group A.

**Table 4:** The prevalence of adverse pregnancy outcomes according to the number of out-of-range lipids in early pregnancy

	N (%)	GDM	OR	Pregnancy-induced hypertension	OR	PE	OR	Macrosomia	OR	LGA	OR	Preterm birth	OR	Total	OR
		N (%)		N (%)		N (%)				N (%)		N (%)		N (%)	
Group A	10,793 (79.1%)	1895 (17.9%)	1	331 (3.1%)	1	170 (1.6%)	1	821 (7.6)	1	1733 (16.1%)	1	442 (4.1%)	1	3754 (34.8%)	1
Group B	2060 (15.1%)	568 (28.1%)	1.567 (1.400–1.753)	129 (6.4%)	1.840 (1.486–2.279)	65 (3.2%)	1.836 (1.367–2.466)	217 (10.5)	1.343 (1.143–1.578)	444 (21.6)	1.358 (1.206–1.530)	105 (5.1%)	1.178 (0.944–1.469)	1006 (48.8%)	1.604 (1.455–1.769)
Group C	662 (4.9%)	193 (29.7%)	1.667 (1.391–1.998)	50 (7.7%)	2.132 (1.551–2.930)	24 (3.7%)	1.966 (1.261–3.006)	59 (8.9)	1.025 (0.771–1.362)	147 (22.2%)	1.310 (1.079–1.592)	40 (6.0%)	1.417 (1.005–1.996)	332 (50.2%)	1.618 (1.376–1.903)
Group D	131 (1%)	50 (38.8%)	2.318 (1.596–3.368)	10 (7.7%)	2.039 (1. 048–3.965)	4 (3.1%)	1.588 (0.576–4.380)	18 (13.7)	1.570 (0.934–2.639)	39 (29.8%)	1.856 (1.261–2.731)	5 (3.8%)	0.898 (0.363–2.217)	78 (59.5%)	2.241 (1.560–3.220)

Abbreviations: *TC* total cholesterol, *TG* triglycerides, *HDL-C* high-density lipid cholesterol, *LDL-C* low-density lipid cholesterol, *GDM* gestational diabetes mellitus, *PE* preeclampsia, *LGA* large for gestational age; Group A, no ‘out-of-range’ lipid profile; Group B, one ‘out-of-range’ lipid; Group C, two ‘out-of-range’ lipids; Group D, three or four ‘out-of-range’ lipids

Adjusted for maternal age, pre-pregnancy body mass index, educational level and gestational age at the time of lipid measurement

**Table 5:** The prevalence of adverse pregnancy outcomes according to the number of out-of-range lipids in middle pregnancy

	N (%)	GDM	OR	Pregnancy-induced hypertension	OR	PE	OR	Macrosomia	OR	LGA	OR	Preterm birth	OR	Total	OR
		N (%)		N (%)		N (%)				N (%)		N (%)		N (%)	
Group A	5245 (81.1%)	1289 (25.0%)	1	207 (4.0%)	1	110 (2.1%)	1	503 (9.6%)	1	991 (18.9%)	1	230 (4.4%)	1	2247 (42.8%)	1
Group B	917 (14.2%)	307 (34.3%)	1.563 (1.343–1.819)	66 (7.3%)	1.616 (1.204–2.168)	25 (2.8%)	1.088 (0.692–1.711)	101 (11.0%)	1.081 (0.857–1.362)	211 (23.0%)	1.186 (0.998–1410)	60 (6.5%)	1.332 (0.986–1.800)	484 (52.8%)	1.325 (1.142–1.536)
Group C	277 (4.3%)	85 (31.1)	1.358 (1.043–1.769)	23 (8.5%)	1.821 (1.120–2.959)	12 (4.4%)	1.503 (0.748–3.022)	22 (7.9%)	0.815 (0.521–1.274)	54 (19.5%)	1.037 (0.762–1.411)	30 (10.8%)	2.217 (1.437–3.421)	141 (50.9%)	1.246 (0.967–1.607)
Group D	27 (0.4%)	5 (18.5%)	0.683 (0.258–1.807)	3 (11.1%)	2.643 (0.778–8.982)	2 (7.4%)	3.158 (0.724–13.776)	2 (7.4%)	0.719 (0.169–3.051)	6 (22.2%)	1.165 (0.467–2.909)	6 (22.2%)	5.782 (2.246–14.885)	14 (51.9%)	1.270 (0.580–2.782)

Abbreviations: *TC* total cholesterol, *TG* triglycerides, *HDL-C* high-density lipid cholesterol, *LDL-C* low-density lipid cholesterol, *GDM* gestational diabetes mellitus, *PE* preeclampsia, *LGA* large for gestational age; Group A, no ‘out-of-range’ lipid profile; Group B, one ‘out-of-range’ lipid; Group C, two ‘out-of-range’ lipids; Group D, three or four ‘out-of-range’ lipids

Adjusted for maternal age, pre-pregnancy body mass index, educational level and gestational age at the time of lipid measurement

## Discussion

The results of the present study revealed that serum levels of TC, TG, LDL-C and HDL-C all increased significantly from early pregnancy to middle pregnancy, with the most prominent features being an elevation of serum TG and, to a lesser extent, elevations of TC, HDL-C and LDL-C. Moreover, a trend towards an increasing incidence of adverse pregnancy outcomes was observed with increasing levels of TC, TG, and LDL-C and decreasing levels of HDL-C in both early and middle pregnancy. Thus, we recommend that the reference values of serum TC, TG and LDL-C in early and middle pregnancy should be less than the 95th percentiles and the reference value of HDL-C should be greater than the 5th percentile. Specifically, in early pregnancy, these values should be TC < 5.64 mmol/L, TG < 1.95 mmol/L, HDL-C > 1.23 mmol/L and LDL-C < 3.27 mmol/L, and in middle pregnancy, they should be TC < 7.50 mmol/L, TG < 3.56 mmol/L, HDL-C > 1.41 mmol/L and LDL-C < 4.83 mmol/L. Furthermore, the more of out-of-range lipids pregnant women had in early and middle pregnancy, the higher their risk of developing adverse pregnancy outcomes.

The changes in maternal lipid concentrations during pregnancy observed in our study were similar to those reported in previous studies that showed that blood lipid concentrations increased during pregnancy, with TG levels changing the most [16, 17]. Changes in serum lipid levels during pregnancy are thought to be affected by hormonal changes, including increases in serum levels of oestrogen and progesterone [18–20]. In addition, hyperinsulinaemia and insulin resistance during pregnancy have significant effects on lipid metabolism and serum levels [18]. In this study, we did not have data on lipid concentrations after pregnancy; however, other studies have indicated that lipid concentrations return to pre-pregnancy concentrations after delivery [2, 21, 22], suggesting that the increase in blood lipids during pregnancy could have an important role in the physiology of the pregnancy and development of the foetus.

Maternal fat accumulation in the first two-thirds of gestation and hyperlipidaemia with increased lipolysis in the third trimester are essential for an adequate nutrient supply for foetal growth and development [3]. For example, foetuses use TC to build cell membranes and as the precursor of bile acids and steroid hormones. It is also required for cell proliferation and development of the growing body. TG serves as an energy depot for maternal dietary fatty acids and contributes significantly to foetal growth and development [23]. HDL-C plays a positive role in protecting the maternal vascular endothelium during pregnancy [24].

However, similar to abnormal glucose metabolism, dyslipidaemia during pregnancy could also adversely affect the intrauterine environment, leading to short- and long-term health issues for both mothers and their offspring. Our current study confirmed a trend towards an increasing incidence of adverse pregnancy outcomes with increasing levels of TC, TG, and LDL-C and decreasing levels of HDL-C in both early and middle pregnancy. These findings agreed with the results of existing studies. Vrijkotte TG et al. found that every unit increase in TG levels during early gestation was linearly associated with an increased risk of hyperglycaemia in pregnancy, preeclampsia, LGA and preterm delivery, suggesting that lifestyle programmes should be conducted in women of reproductive age with a focus on lowering triglyceride levels [16]. Moreover, an increase in TC and LDL-C levels during pregnancy is also considered a risk factor for GDM, preterm delivery and PE [9, 25, 26]. By contrast, elevation in HDL-C levels is associated with a decreased risk of GDM, macrosomia and PE and was thought to be a protective factor for both ourcomes [4, 6, 8].

Thus, based on the outcomes of our analysis and those of others, we recommend that the reference values of serum TC, TG and LDL in early and middle pregnancy should be less than the 95th percentiles and the reference value of HDL should be greater than the 5th percentile. To date, few studies have reported reference ranges that can be used to evaluate the results of lipid measurements in women during pregnancy. Therefore, obstetricians are often in doubt as to whether lipid levels are ‘normal’ for a given period of pregnancy. By examining 719 healthy pregnant women, 172 in the first trimester, 227 in the second trimester and 320 in the third trimester, Piechota W. et al. similarly proposed that TC, TG and LDL-C levels exceeding the 95th percentile should be used to define underlying hyperlipidaemia, and HDL-C levels below the 5th percentile should be regarded as abnormally low. In their study, all lipids were significantly elevated during the second and the third trimesters with the most prominent change being a 2.7-fold increase in TG levels in the third trimester. The reference ranges established in the second and third trimesters were as follows: TC: < 8.24 and < 9.83 mmol/l; TG: < 2.87 and < 4.68 mmol/l; LDL-TC: < 5.61 and < 6.48 mmol/l; and HDL: > 1.09 and 1.04, respectively [27]. Earlier, Knopp RH et al. attempted to report the reference ranges that could be used to evaluate the results of lipid measurements in women during pregnancy. Although their study was restricted to women at 36 weeks of gestation, the reference values in that study were based on the following 95th percentiles of the distributions: TC, 318 mg/dl; TG, 387 mg/dl; and LDL-C, 218 mg/dl. The fifth percentile for HDL-C was 42 mg/dl [28]. However, because few studies have focused on this issue, and because none of the existing studies have contained sufficient numbers with good quality, it remains impossible to extract a set of typical lipid values for the different periods of pregnancy. Furthermore, the normal range of blood lipids during pregnancy should also vary according to ethnic groups. Nevertheless, the most common criteria used as a reference for lipids during pregnancy referred to “Williams Obstetrics—24th Edition” [29]. The reference ranges recommended are: 1st trimester: TC 141–210 mg/dl, TG 40–159 mg/dl, HDL-C 40–78 mg/ml, and LDL-C 60–153 mg/ml; 2nd trimester: TC 176–299 mg/dl, TC 70–382 mg/dl, HDL-C 52–87 mg/ml, and LDL-C 77–184 mg/ml; and 3rd trimester: TC 219–349 mg/dl, TC 131–453 mg/dl, HDL-C 48–87 mg/ml and LDL-C 101–224 mg/ml. Notably, based on the results of our present analysis and one previous study [6], we demonstrated that high levels of TC, TG, and LDL-C and low levels of HDL-C may be predictive biomarkers for adverse pregnancy outcomes, whereas in early pregnancy, low TC, TG, and LDL-C levels and high HDL-C levels could have some protective roles. Therefore, we did not set low cut-offs for TC, TG and LDL-C or a high cut-off for HDL-C. The high cut-offs for TC, TG and LDL-C and the low cut-off for HDL-C proposed in the present study were similar to those recommended in “Williams Obstetrics—24th Edition”.

In the present analysis, the associations between TG or HLD-C and adverse pregnancy outcomes appeared to be stronger than the associations between TC or LDL-C and adverse pregnancy outcomes, particularly during middle pregnancy. Therefore, in practice, pregnant women with out-of-range values for TG or HDL-C might have a greater risk of developing adverse pregnancy outcomes than those with out-of-range values for TC or LDL-C. Moreover, for the reference ranges presented in this study to have greater clinical and research significance, we further examined whether the number of out-of-range lipids in pregnant women was logical. Not surprisingly, the more out-of-range lipids, the greater the risk of developing adverse pregnancy outcomes. However, the incidence rate of GDM appeared to decrease as the number of out-of-range lipids increased, although this relationship was not significant. We hold the opinion that it might be due to the women’s lifestyle interventions. In our study design, we defined middle pregnancy as 14 ≤ gestational weeks < 28, and GDM was diagnosed during this interval; therefore, the lifestyle interventions could have an effect on the original correlation between lipids and the incidence of GDM. Lifestyle interventions, including dietary changes and exercise, have shown efficacy in modifying abnormal lipid levels [30, 31]. This is why we only recommend the reference ranges available for maternal blood lipid concentrations in early and middle pregnancy. Lifestyle interventions are more likely to occur in the latter half of the pregnancy period than during the first half of pregnancy. Conversely, the purpose of defining reference ranges for maternal blood lipids was to identify high-risk groups and to conduct appropriate interventions in a timely fashion to reduce adverse pregnancy outcomes. Therefore, the reference ranges in early and middle pregnancy appear to be more meaningful.

To the best of our knowledge, our study is one of the few studies conducted in the world and the first in China to report reference ranges that can be used to evaluate the results of lipid measurements in women during the various periods of pregnancy. In addition, by analysing the number of out-of-range lipids pregnant women had and their risk of adverse pregnancy outcomes, we believe the reference values we recommended are much more practical in clinical work. This study was rationally designed and was conducted by trained staff; moreover, most of the data collected were obtained from medical records. Therefore, there was an assurance of the standardization of data collection. Furthermore, compared to previous studies, ours had a relatively sufficient number of samples, even though we excluded many participants because blood lipid tests are not currently included in routine testing during pregnancy in China. Nevertheless, the risk of selection bias was inevitable. In addition, several other limitations of this study should be noted. First, because of the retrospective nature of our study, it was impossible for us to eliminate all confounders that affected the associations between maternal blood lipids and adverse pregnancy outcomes. Second, there may be natural correlations between p-BMI and lipids themselves. Thus, our analysis and adjustment for p-BMI could weaken the correlations between lipid profiles and adverse pregnancy outcomes. Third, we focused on Chinese singleton pregnant women; therefore, our results may not be generalizable to the overall population.

## Conclusion

In conclusion, there were trends towards an increasing incidence of adverse pregnancy outcomes with increasing levels of early pregnancy TC, TG and LDL-C, and a decreasing level of HDL-C during the first and second trimesters. Therefore, we recommend that the reference values of serum TC, TG and LDL in early and middle pregnancy should be less than the 95th percentiles, and that of HDL should be greater than the 5th percentile. Furthermore, the higher the number of out-of-range lipids (high TC, TG and LDL-C levels and low HDL-C levels) a woman had, the higher her risk of developing adverse pregnancy outcomes. Thus, it is necessary to pay attention to maternal lipid values during pregnancy. We believe that our study provides more evidence to support lipid screening during pregnancy. Additional, well-designed epidemiological studies with larger sample sizes are necessary to determine more accurate normal lipid reference values. Of course, if the reference values are determined with consideration of the changes in blood lipids during pregnancy, it would be more valuable and accurate.

## Abbreviations

CIs: confidence intervals
GDM: gestational diabetes mellitus
HDL-C: high-density lipid cholesterol
LDL-C: low-density lipid cholesterol
LGA: large for gestational age
ORs: odds ratios
p-BMI: pre-pregnancy body mass index
PE: pre-eclampsia
PUFH: Peking University First Hospital
TC: total cholesterol
TG: triglycerides
